# Nucleation of Ga droplets self-assembly on GaAs(111)A substrates

**DOI:** 10.1038/s41598-021-86339-3

**Published:** 2021-03-25

**Authors:** Artur Tuktamyshev, Alexey Fedorov, Sergio Bietti, Stefano Vichi, Riccardo Tambone, Shiro Tsukamoto, Stefano Sanguinetti

**Affiliations:** 1 Department of Material Science, University of Milano-Bicocca, via R. Cozzi 55, 20125 Milan, Italy; 2 CNR Istituto di Fotonica e Nanotecnologie, Piazza Leonardo da Vinci 32, 20133 Milan, Italy; 3 Laboratory for Nanostructure Epitaxy and Spintronics on Silicon, Polo di Como, via F. Anzani 42, 22100 Como, Italy

**Keywords:** Materials science, Nanoscience and technology, Physics

## Abstract

We investigated the nucleation of Ga droplets on singular GaAs(111)A substrates in the view of their use as the seeds for the self-assembled droplet epitaxial quantum dots. A small critical cluster size of 1–2 atoms characterizes the droplet nucleation. Low values of the Hopkins-Skellam index (as low as 0.35) demonstrate a high degree of a spatial order of the droplet ensemble. Around $350\,^{\circ }\hbox {C}$ the droplet size distribution becomes bimodal. We attribute this observation to the interplay between the local environment and the limitation to the adatom surface diffusion introduced by the Ehrlich–Schwöbel barrier at the terrace edges.

## Introduction

The unique properties of the self-assembled quantum dots (QDs), such as the discrete energy levels and a precise control of additional features, like entangled photon emission, by the QD shape and size, have a great potential in the optoelectronic device fabrication for the future quantum network applications^1–4^. For this reason, one of the main challenges in the QD self-assembly is the reproducibility of QDs in terms of shape and size.

Droplet epitaxy (DE) is well-established for the formation of III-V compound semiconductor nanostructures and allows to control the QD density and size in a wide range^5,6^. The size distribution of the self-assembled DE QDs is strictly determined by the original size distribution of the droplets^7^. As the droplet size distribution can be easily controlled, using DE technique, it is then possible to obtain a narrow QD size distribution, resulting in a small ensemble photoluminescence linewidth^8^.

The (111)-oriented surfaces show a C$_{3v}$ symmetry, which allows to self-assemble highly symmetric QDs. These are necessary to reduce the fine structure splitting of the exciton state for the generation of highly entangled photons^9–11^. The QD self-assembly on (111) surfaces is complex using the Stranski-Krastanov (SK) growth mode^12^ (only recently the self-assembly of SK QDs on the (111)A surface was demonstrated^13,14^ by taking profit of tensile strain). Nevertheless, DE allows the density and the size control of the self-assembled QDs on (111)A surfaces^11,15^. Recently, the studies of shape-controlled highly symmetric DE GaAs QDs grown on AlGaAs/GaAs(111)A^11^ for entangled photon pair generation^10^ have been published. An additional interest to (111)A face is related to the possibility to self-assemble DE QDs without the formation of the wetting layer under the QD layer, since GaAs(111)A surface is in Ga-rich conditions and the deposition of group III atoms on the surface leads to immediate nucleation of the liquid droplets.

The epitaxial growth on (111) surfaces is complicated because the surface morphology is affected by the growth conditions. Nevertheless, using low growth rate and high V/III flux ratio for the growth of GaAs and AlGaAs layers on GaAs(111)A, it is possible to suppress an amount of hillocks nucleated by the stacking faults^16^ to improve the crystalline quality of the epitaxial layers.

In this work, we investigated the Ga droplet self-assembly on singular GaAs(111)A substrates in order to gain fundamental understanding of the effects of the surface characteristics, in terms of a surface reconstruction and a morphology, on the island nucleation dynamics. The droplet density dependence on the temperature has been determined in the temperature range between 300 and $450\,^{\circ }\hbox {C}$. This gave us access to the fundamental physical quantities which determine the droplet formation dynamics: (1) the critical nucleus size for the droplet formation^17,18^; (2) the adatom surface diffusivity and its dependence on the surface reconstruction. We have also investigated the effect of surface defects on the droplet size distribution in combination with the presence of the sizeable Ehrlich–Schwöbel barrier typical for the GaAs(111)A surface^16^.

## Methods

The samples were grown, using a molecular beam epitaxy (MBE) system, on an undoped singular GaAs(111)A substrates. The substrate temperature was measured by the thermocouple situated between the substrate heater and the sample, and by the infrared pyrometer.

After an oxide desorption at $590\,^{\circ }\hbox {C}$, a 50 nm GaAs buffer layer was deposited at the temperature of $520\,^{\circ }\hbox {C}$ with a deposition rate of 0.07 ML/s (here and below 1 ML is defined as $6.26\times 10^{14}\,\hbox {atoms/cm}^{2}$, which is the site-number density of the unreconstructed GaAs(001) surface), in order to obtain a smooth surface^16^. The substrate temperature was then decreased to the droplet deposition temperature varying from 300 to $450\,^{\circ }\hbox {C}$. During the droplet deposition the As cell valve was closed in order to deplete the growth chamber from the arsenic molecules. 2 ML of gallium were deposited at a rate of 0.01 ML/s. During the Ga deposition, the background pressure was below 3$\times$10$^{-9}$ torr. The supply of Ga without As$_4$ enabled the appearance of the Ga liquid droplets on the buffer layer surface. Next, an As$_4$ flux with a beam equivalent pressure (BEP) of $6.2\times 10^{-5}$ torr was supplied at the same temperature for 3 minutes, in order to crystallize Ga droplets into GaAs islands. The sample T2b was arsenized after 30 minutes of annealing of Ga droplets at $350\,^{\circ }\hbox {C}$, in order to study the influence of the ripening processes on the island density and size.

As shown in Refs.^19–21^, the density of DE QDs, when crystallized at the same temperature used for the deposition of the metal droplets, mirrors that of droplets, thus making possible to access the density dependence of Ga droplets on the deposition parameters through the measurement of the island density. The crystallized surface morphology is more stable on time and avoids the strong oxidation effects which would hinder the reproducibility of the atomic force microscope (AFM) measurements.

The ex-situ morphological characterization of the samples was performed by AFM in a tapping mode, using tips capable of a lateral resolution of about 2 nm.

The growth description of the samples is summarized in Table 1.

**Table 1:** The substrate temperature during the Ga droplet nucleation and the subsequent arsenization of the samples, presented in this work as well as the GaAs island density of the samples.

Sample	$T,^{\circ }\hbox {C}$	$N,\,\hbox {cm}^{-2}$	Comments
T1	300	($7.67\pm 1.15)\times 10^{10}$	
T2	350	($1.83\pm 0.06)\times 10^{10}$	
T2b	350	($1.85\pm 0.09)\times 10^{10}$	Arsenization after 30 minutes annealing
T3	400	$(2.27\pm 0.05)\times 10^{9}$	
T4	450	$(7.17\pm 1.08)\times 10^{8}$	

## Results and discussion

**Figure 1:**
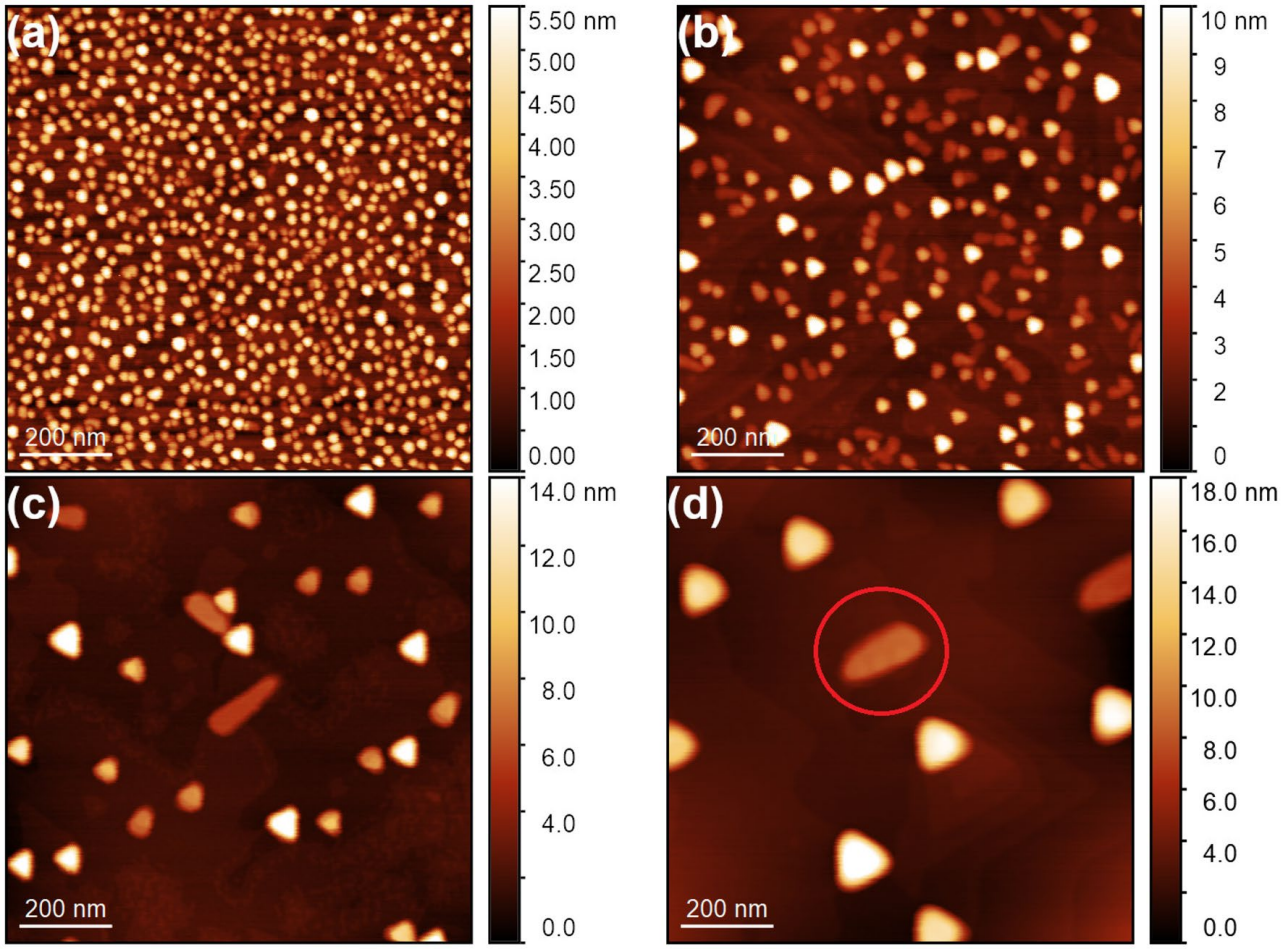
AFM topography images of GaAs islands grown on the GaAs(111)A substrate at **(a)** $300\,^{\circ }\hbox {C}$ ($1\times 1\,\upmu \hbox {m}^{2}$, sample T1); **(b)** $350\,^{\circ }\hbox {C}$ ($1\times 1\,\upmu \hbox {m}^{2}$, sample T2); **(c)** $400\,^{\circ }\hbox {C}$ ($1\times 1\,\upmu \hbox {m}^{2}$, sample T3); **(d)** $450\,^{\circ }\hbox {C}$ ($1\times 1\,\upmu \hbox {m}^{2}$, sample T4). The red circle highlights a kinked island.

Figure 1 shows AFM images of the samples T1, T2, T3, and T4 with GaAs islands, grown at 300, 350, 400, and $450\,^{\circ }\hbox {C}$, respectively. The GaAs islands grown at $300\,^{\circ }\hbox {C}$ show a hexagonal shape. With the increasing the crystallization temperature the shape of islands becomes triangular. This observation in agreement with previous reports and it is related to the crystallization kinetics of the islands^11,15^. As expected, with increasing the deposition temperature the island density decreases^19–22^. This tendency satisfies the classical nucleation theory of Venables^17,18^, which describes a nucleation of Ga droplets as a thermally activated process. As a result, the density of droplets *N* is reproduced by the Arrhenius law:

1 $$\begin{aligned} N \propto exp(E_{a}/k_{B}T), \end{aligned}$$

where $E_{a}$ is the nucleation activation energy, $k_{B}$—the Boltzmann’s constant, and *T* is the Ga droplet deposition temperature.

It is worth to mention that some of DE GaAs islands, grown on a singular GaAs(111)A surface, are “kinked” (Ga droplets are spontaneously crystallized in a horizontal direction). One kinked dot is highlighted on Fig. 1d. The analogues of such islands are kinked nanowires (NWs) performed by the vapor-liquid–solid (VLS) growth on (111) surfaces^23–25^. The growth in a horizontal direction and a subsequent formation of kinked NWs is induced by a twin-mediated mechanism, which can be suppressed/maintained by controlling the growth conditions^24,25^. This behavior of a spontaneous nucleation of kinked GaAs islands on singular GaAs(111)A surface is in agreement with the expected island formation during the crystallization (arsenization) process of Ga droplets under the VLS mechanism.

**Figure 2:**
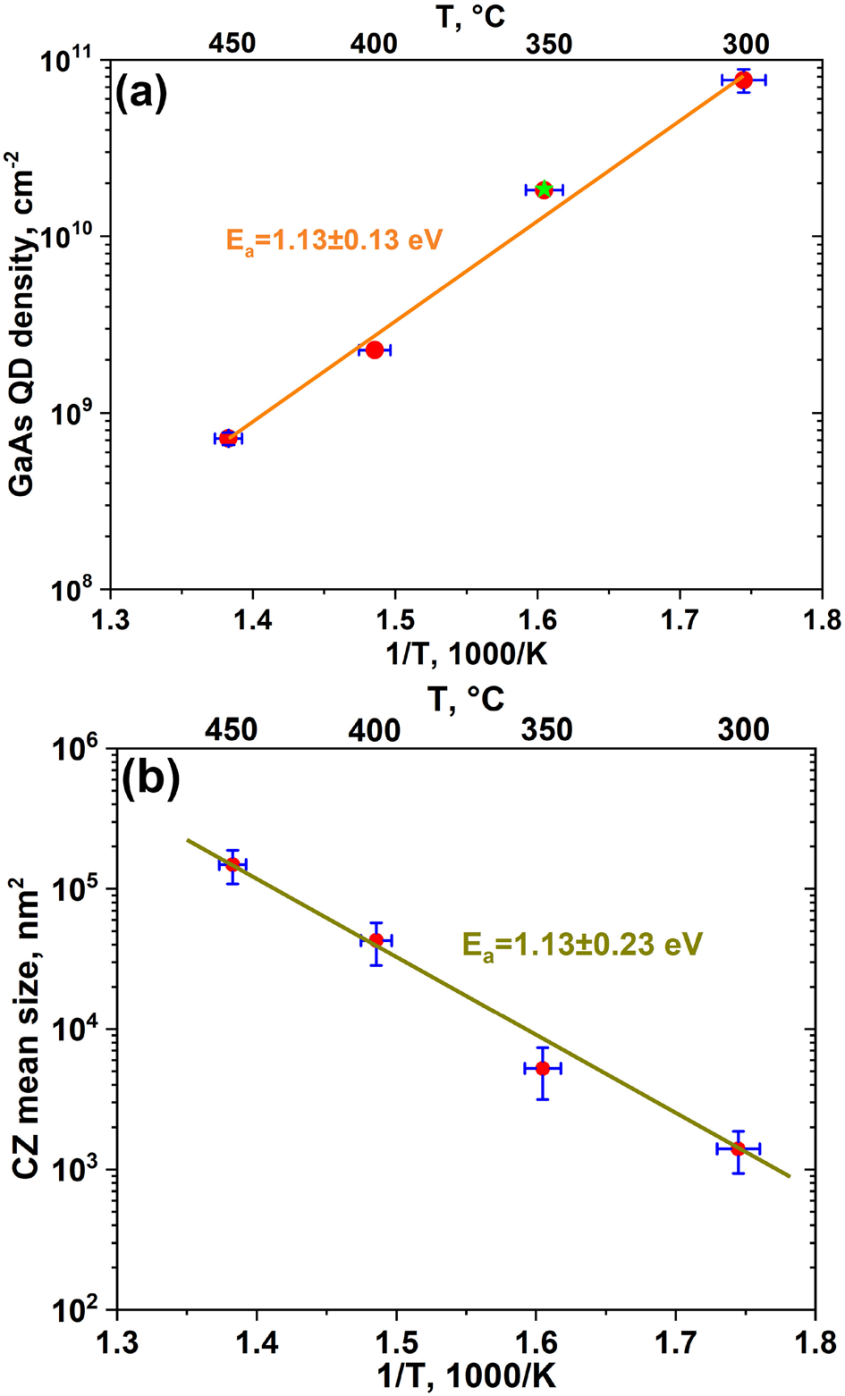
**(a)** The temperature density dependence of DE GaAs islands grown on GaAs(111)A substrate (red points). The green star indicates the island density of the sample T2b. The nucleation activation energy, $E_{a}~=~1.13\pm 0.13~\hbox {eV}$. **(b)** The temperature dependence of a mean size of Voronoi cells. The activation energy $E_{a}$, calculated from this method, equals 1.13±0.23 eV. The temperature error bar for both graphs is $\pm 5\,^{\circ }\hbox {C}$.

Using the Arrhenius plot of the density dependence on the deposition temperature, it is possible to calculate the activation energy $E_{a}$. Figure 2a shows the temperature density dependence of GaAs islands in the range of 300–$450\,^{\circ }\hbox {C}$. The temperature error bar in our measurements is associated with the accuracy of the substrate temperature determination by the thermocouple and equals roughly $\pm 5\,^{\circ }\hbox {C}$. The density calculation was carried out using the data of several AFM scans from different areas of the samples. The density ranges from $7\times 10^{8}\,\hbox {cm}^{-2}$ to $8\times 10^{10}\,\hbox {cm}^{-2}$. The activation energy, calculated from the temperature dependence of the island density, is $1.13\pm 0.13\,\hbox {eV}$. This value is in between the ones, which we calculated for the Ga droplet nucleation on vicinal GaAs(111)A^21^. It is assumed that the the Ga droplet nucleation on singular GaAs(111)A is characterized by both types of diffusion (one- and two-dimensional).

A drastic change in the slope of the Arrhenius plot at *T* >$200\,^{\circ }\hbox {C}$ was observed previously for the temperature density dependence of DE GaAs^19^ and islands grown on GaAs(001) and Ga droplets on singular GaAs(111)A^22^. Such a phenomenon was attributed to the onset of the Ostwald ripening process (the growth of large clusters on the cost of smaller ones and hence a decrease of the total cluster density in a closed thermodynamic system^26^) with increasing the deposition temperature. The change in the density of DE InAs QDs on GaAs(001) grown by metal-organic chemical vapor deposition was also associated with the Ostwald ripening during the nucleation of In droplets^27^. In the present work we did not observe such a change of the Arrhenius slope. In order to check the presence of the Ostwald ripening, we prepared the sample T2b, for which the arsenization process was carried out after 30 minutes of an annealing process at the same temperature. The island density of the sample equals $(1.85\pm 0.09)\times 10^{10}~\hbox {cm}^{-2}$ (the green star on Fig. 2a). The value is only slightly higher than that observed in the sample T2 - $(1.83\pm 0.06)\times 10^{10}\,\hbox {cm}^{-2}$, for which the annealing was not performed. This suggests that no Ostwald ripening occurs after the deposition of Ga droplets in our experiments.

Additionally, the characteristic parameters of the nucleation process such as the activation energy $E_{a}$ and the size of stable cluster *i* (the number of atoms that are the part of the largest unstable cluster^17,18^) can be obtained from the capture zone distribution (CZD) approach^21,28,29^. The capture zone (CZ) is the area from which, on average, the adatom constituting the island are collected. The exact shape of the normalized distribution of CZs strictly depends on the processes at the basis of the nucleation process^28^. The CZs can be determined from the Voronoi approach, a particular case of a surface tessellation where, given a set of centers (in our case the droplets), the space is divided according to their “area of influence”. In fact, the Voronoi cell identifies the area of the surface that is closer to the cell center respect to any other in the ensemble. CZD analysis provides an information about the size of the critical cluster and reflects the dimensionality of the nucleation process and can be extended to any aggregation-limited process^29^. Using this approach, the critical cluster size can be directly obtained from AFM scans and compared to the value, estimated from the droplet density dependence on the flux rate.

Figure 2b shows the temperature dependence of a mean size of Voronoi cells for the samples T1, T2, T3, and T4. Naturally, the total number of Voronoi cells are related to the island density, so using this dependence, it is also possible to determine $E_{a}$. From the CZD approach $E_{a} = 1.13\pm 0.23\,\hbox {eV}$, which is in agreement with the value determined from the island density dependence.

**Figure 3:**
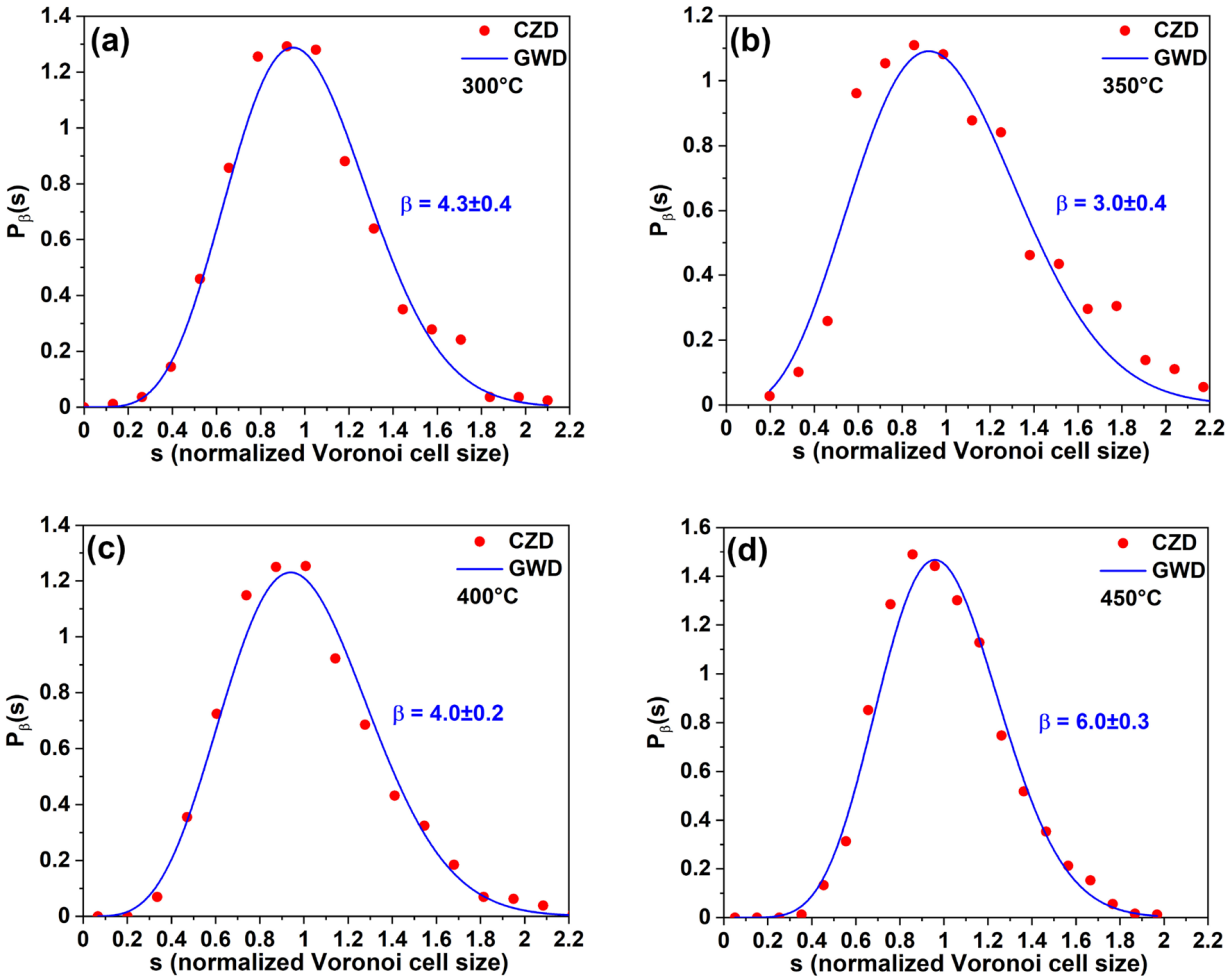
CZDs of the samples **(a)** T1, **(b)** T2, **(c)** T3, and **(d)** T4, fitted by the GWD.

In order to determine the critical cluster size *i*, we analyzed normalized (to the mean size of Voronoi cells) CZD from the Voronoi tessellation. The predicted analytical form of CZD coincides with generalized Wigner distribution (GWD), proposed for the nearest-neighbor spacing distribution^21,28,29^ (see Fig. 3):

2 $$\begin{aligned} P_{\beta }(s)=a_{\beta } s^{\beta } exp(-b_{\beta } s^{2}), \end{aligned}$$

where s is the capture zone (CZ) area divided by its average value, $\hbox {a}_{\beta }$ and $\hbox {b}_{\beta }$ are the constants that assure the normalization and the unit mean conditions, respectively.

The fitting parameter $\beta$ depends on *i* and the dimensionality of the diffusion $\gamma$ as^21,29^

3 $$\begin{aligned} \beta = \gamma i + \gamma + i. \end{aligned}$$

**Table 2:** The fitting parameter $\beta$ and the critical cluster size *i* of the samples T1, T2, T3, and T4.

Sample	$\beta$	$i = \beta - 2$
T1 ($300\,^{\circ }\hbox {C}$)	$4.3\pm 0.4$	$2.3\pm 0.4$
T2 ($350\,^{\circ }\hbox {C}$)	$3.0\pm 0.4$	$1.0\pm 0.4$
T3 ($400\,^{\circ }\hbox {C}$)	$4.0\pm 0.2$	$2.0\pm 0.2$
T4 ($450\,^{\circ }\hbox {C}$)	$6.0\pm 0.3$	$4.0\pm 0.3$

The parameter $\beta$ and the critical cluster size *i* obtained with $\gamma$ = 1 (taking into account a two-dimensional adatom diffusion^21,29^) are presented in Table 2. For $\hbox {T} \le 400^{\circ }\hbox {C}$ the critical cluster size for the Ga droplet nucleation on singular GaAs(111)A *i* = 1–2 atoms, which is in good agreement with the previous studies^21,22^. We observed an increase of the critical cluster size at $450^{\circ }\hbox {C}$, which is compatible with the expected stable cluster decomposition due to the thermally activated atom detachment processes^17,18^.

**Figure 4:**
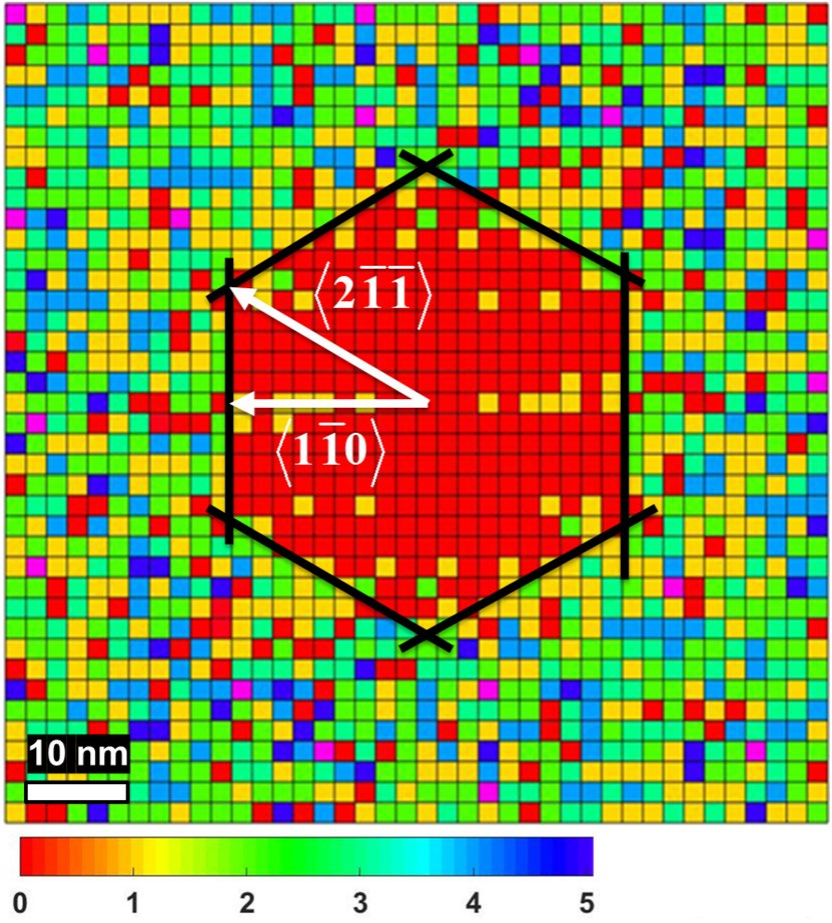
Island-island distance distribution histogram showing the spatial neighbor distribution of GaAs islands for the sample T1 (80$\times$80 nm$^{2}$). The bin value color code is indicated.

The diffusivity behavior of Ga adatoms can be monitored through the spatial neighbor distribution of islands^21^. In Fig. 4 we report the two-dimensional histogram of the island-island relative distance $\mathbf {R}_i-\mathbf {R}_j$, where $\mathbf {R}_i$ and $\mathbf {R}_j$ indicate the position of two different islands, for sample T1. The rest of the samples show similar distribution, but with lower statistics, since the island density is higher at lower deposition temperature. The histogram shows that there is, on average, an excluded area (the area without the neighboring islands, highlighted by black lines) around each island with an almost symmetrical, nearly hexagonal, shape with the vertices in the $<2\,\overline{1}\,\overline{1}>$ directions. The shape of the excluded zone suggests that $<2\,\overline{1}\,\overline{1}>$ directions are preferable for the Ga adatom diffusion on GaAs(111)A. This observation is in agreement with the Ga-vacancy (2$\times$2) surface reconstruction of GaAs(111)A^30,31^. According to Ref.^31^, the diffusion activation energies $E_{d}$ equal 1.06 and 1.14 eV for Ga adatom diffusion in the $<2\,\overline{1}\,\overline{1}>$ and $<1\,\overline{1}\,0>$ directions, respectively, on the GaAs(111)A-(2$\times$2) surface reconstruction. Thus, the diffusion length in the $<2\,\overline{1}\,\overline{1}>$ directions should be longer, which we observed from the excluded zone area.

**Figure 5:**
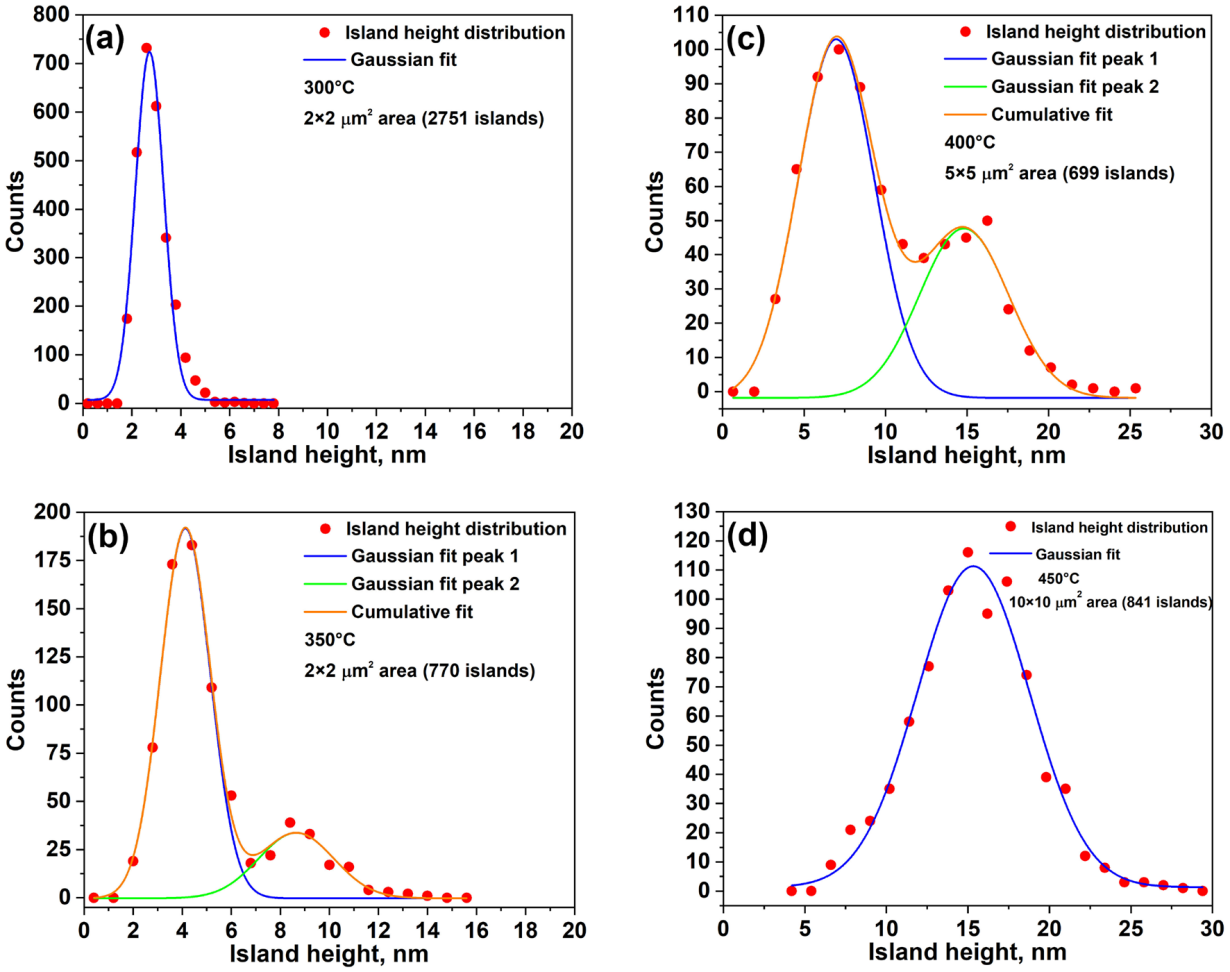
The GaAs island height distribution of samples **(a)** T1, **(b)** T2, **(c)** T3, and **(d)** T4. For the samples T1 and T4 an unimodal distribution is observed with a standard deviation of about 44%. The samples T2 and T3 have a bimodal distribution.

The last goal of this work is to study a size distribution of the grown islands. Figure 5 shows the GaAs island height distribution of the samples T1, T2, T3, and T4 fitted by the Gaussian line shapes. At the low deposition temperature of $300\, ^{\circ }\hbox {C}$ the size distribution has a mean island height of about 2.7 nm with a standard deviation of 43% (Fig. 5a). Similar situation at the high temperature of $450\,^{\circ }\hbox {C}$ (Fig. 5d). The mean height for the sample T4 is about 15.3 nm with the standard deviation of 45%. And the most intriguing observation is the presence of a bimodal island size distribution at the intermediate temperatures ($350-400\,^{\circ }\hbox {C}$). The sample T2 (Fig. 5b) has two groups of islands with the mean heights of 4.1 and 8.7 nm. And the mean island heights for sample T3 (Fig. 5c) are 7.0 and 14.8 nm. For both cases the aspect ratio R (the height over the base) of bigger islands is approximately two times larger than the one of smaller islands.

The most known systems, where the bimodal QD size distribution was observed, are SK InAs/GaAs(001) QDs^32–35^, where the bimodal behavior was described in terms of InAs coverage^33,34^, which is the distinct threshold for the SK dot formation, and SK Ge/Si(001) islands^36,37^, for whom the morphological shape transition of Ge islands is responsible for the bimodal distribution.

In order to study the origin of the bimodal size distribution in our samples, we investigated the degree of order of the droplet spatial arrangement which can be determined via the Hopkins-Skellam index ($I_{HS}$) of the droplet ensemble^38,39^. $I_{HS}$ permits a precise measurement of a spatial regularity of the droplet distribution through the comparison with a purely random spatial distribution of the ensemble elements. $I_{HS}$ is defined as

4 $$\begin{aligned} I_{HS} = \frac{\sum _{i=1}^N r^2_{1i}}{\sum _{i=1}^N r^2_{2i}}, \end{aligned}$$

where *N* is the total number of droplets. The term $r^2_{1i}$ represents the distance to the nearest droplet from a random location in the studied area. Similarly, $r^2_{2i}$ denotes the distance to the nearest droplet from the *i*-th droplet. Therefore, for a completely random distribution (Poisson) of droplets, $I_{HS}$ = 1. A droplet pattern which shows a clusterization returns a value $I_{HS}$ > 1. The minimum value of $I_{HS}$ is obtained for a perfectly ordered pattern of droplets, such as the hexagonal lattice, which gives $I_{HS}$ = 0.14. Therefore, for the patterns with an intermediate regularity, the value of $I_{HS}$ is expected to be framed between 0.14 to 1, with the $I_{HS}$ value increasing with the disorder.

**Figure 6:**
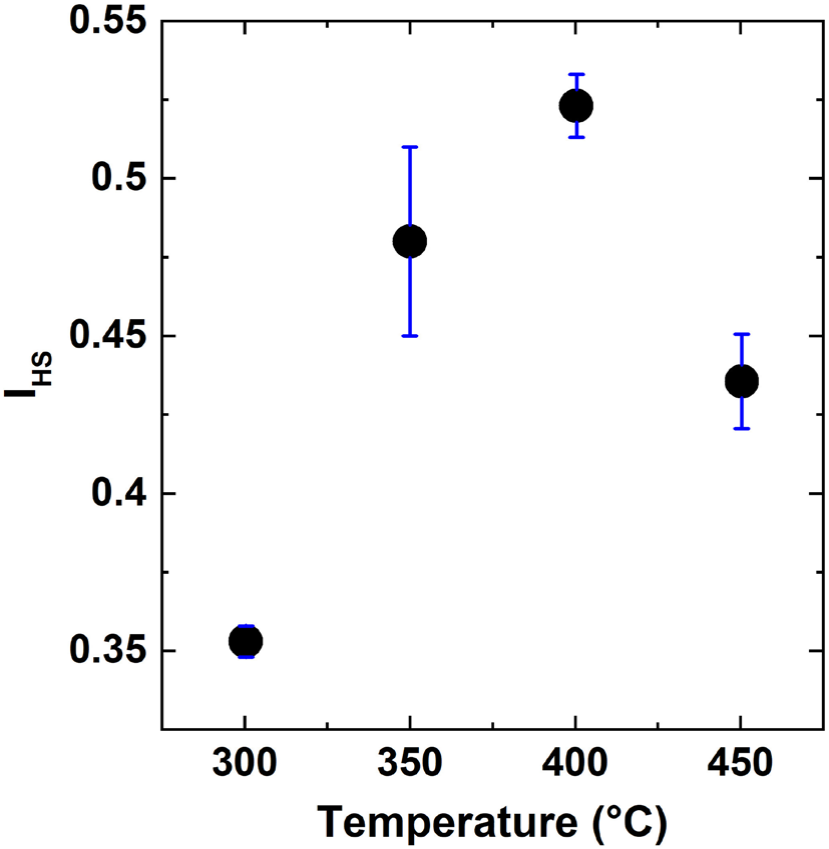
The dependence of the Hopkins–Skellam index ($I_{HS}$) on the deposition temperature.

The dependence of $I_{HS}$ on the deposition temperature is reported in Fig. 6. At low temperatures, the droplets, although randomly located, show a high degree of order, with $I_{HS}$ = 0.35, thus closer to a regular lattice than to a random ensemble. As the temperature reaches the onset of the bimodal distribution (T = $350\,^\circ \hbox {C}$), the Hopkins-Skellam index steeply increases to $I_{HS} \approx 0.5$. A marked dependence on the position on the sample is also observed. The droplet distribution regains a partial order only at $\hbox {T} = 450\,^\circ \hbox {C}$, where $I_{HS} = 0.43\pm 0.01$. Thus, the presence of the bimodal distribution of size walks together with a sizeable decrease of a droplet spatial order, which depends on the location on the sample surface.

As already mentioned, we do not observe ripening processes of Ga droplets. Therefore, the bimodal behavior at the intermediate temperature should stems from a different process able to produce multimodal and broad island size distribution. Together with the Hopkins-Skellam analysis of the spatial arrangement of the droplets, this calls for the presence of a temperature activated extrinsic nucleation process, sensible to the local environment, and affecting the growth kinetics of the droplets in the 350–$400\,^{\circ }\hbox {C}$ temperature range only.

**Figure 7:**
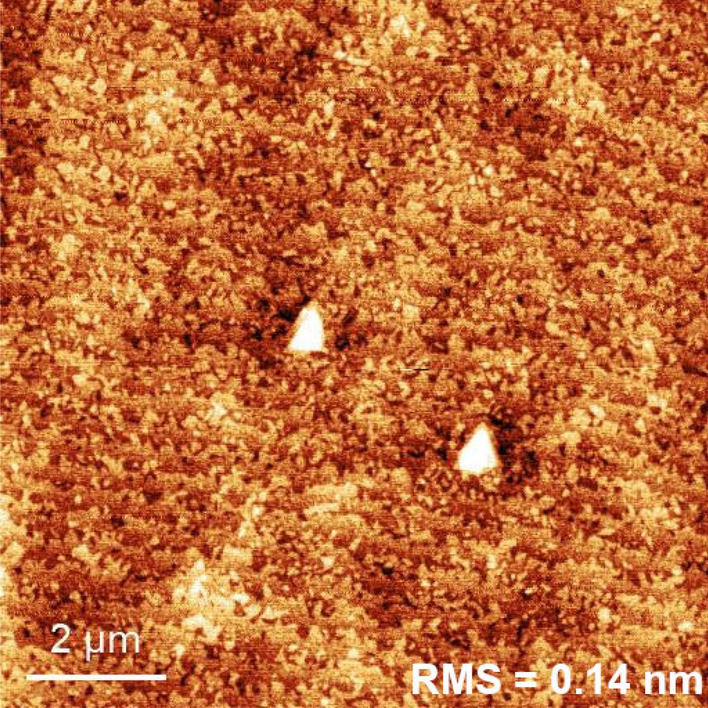
$10\times 10\, \upmu \hbox {m}^{2}$ AFM tapping amplitude image of GaAs buffer layer morphology, grown according to Ref.^16^ and used in this work.

**Figure 8:**
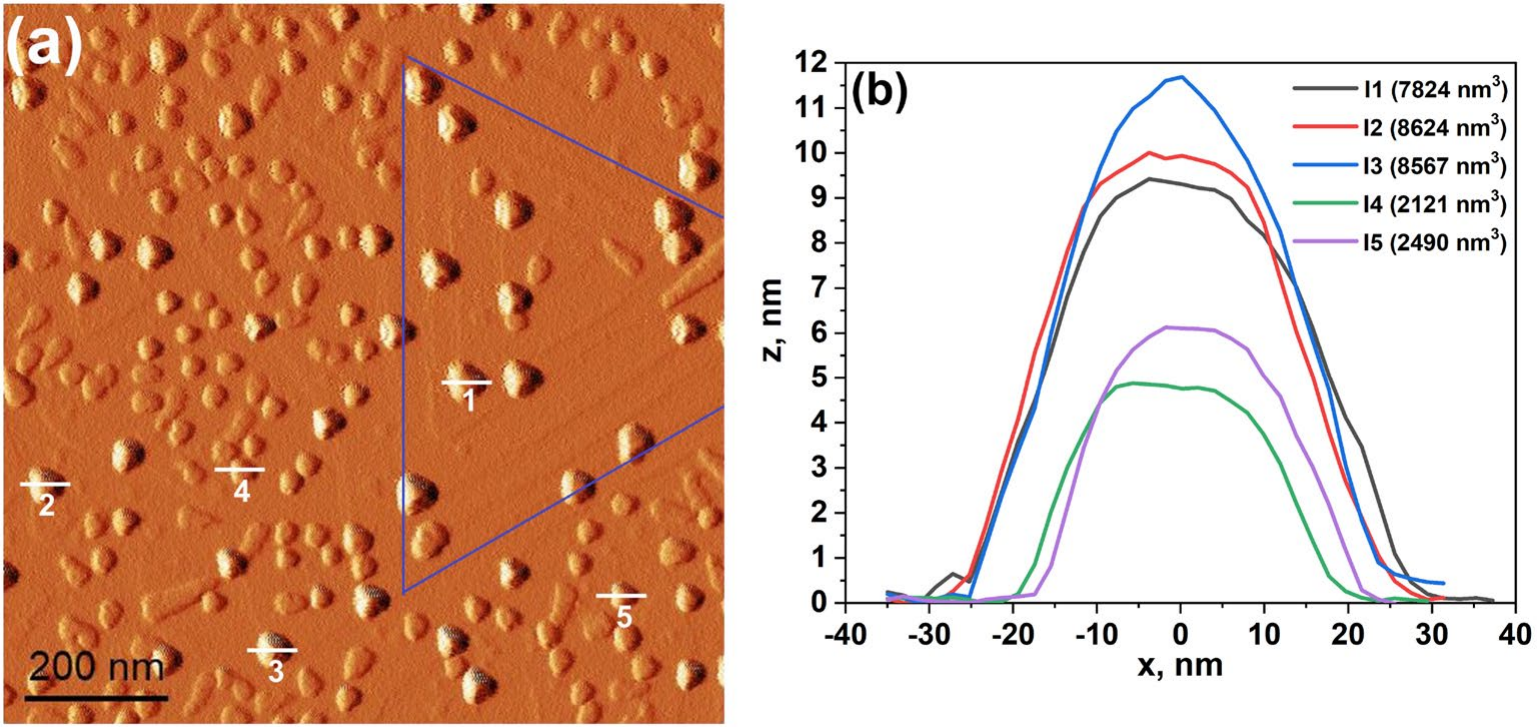
**(a)** $1\times 1\,\upmu \hbox {m}^{2}$ AFM tapping amplitude image of the sample T2. The white lines indicate where the height profiles of islands were measured. The blue lines indicate the area of the stepped hillock. **(b)** The height profiles of 5 islands of the sample taken from **(a)**.

A highly flat and smooth surface was obtained using rather low growth rate and high V/III ratio following the prescription of Ref.^16^. This prevents the formation of large (with $\upmu \hbox {m}$ lateral dimensions) triangular pyramidal hillocks consisting of up to several tens of steps, nucleated by the staking faults^40^. The surface morphology of our samples before the droplet deposition is presented on Fig. 7. The surface is very smooth with a root-mean-square (RMS) roughness of only 0.14 nm. Nevertheless, small hillocks consisting of few steps are still present (see e.g. Fig. 8a, where the stepped triangular pyramid with 4–5 steps is observable inside the area highlighted with the blue lines). The hillocks, thus, introduce a sizeable density of steps on an otherwise nearly free singular GaAs(111)A surface.

We then analyzed the local surface morphology for different island size in the sample T2 (see Fig. 8a). The height profiles of five randomly chosen islands are presented in Fig. 8b. The maximum height of islands I1–I3 (which belong to the large droplet mode) is about 9–12 nm. On the contrary the maximum height of the small islands I4–I5 is only 5–6 nm. Taking also into account the slight change in island radius, the big islands show a volume which is roughly four times larger than the one of the small islands. By analyzing the local environment of the islands, we found that the islands originated from the small droplets which are nucleated within flat terrace areas. On the contrary, the large islands from the large droplets are always nucleated at the step edges joining two terraces. It was shown that the surface step is a preferred nucleation site for In droplet formation on GaAs(001)^41^.

The original surface morphology affects on the droplet size distribution and thus, in turn the island size. A change in the local island density is also observed. The two observations are strictly linked, as an increase in size requires a larger CZ and thus a smaller density. Such local variations of the density, which are restricted to the hillock areas, introduce an additional source of noise in the determination of the ensemble parameters like the nucleation activation energy $E_{a}$ and the critical cluster size *i*. However, this effect appears to be small as it does not hinder the correct determination of the two parameters.

Combining the local dependence of the island size and density with the finite temperature range where the bimodal distribution is observed, we can interpret the relationship between the local environment and the droplet size as stemming from the combined effect of the initial droplet nucleation site and the influence of the presence of ES barrier at the step edges on the adatom diffusion length^16,21,42^.

As a matter of fact, the droplets grow by capturing adatoms roughly within a diffusion length distance from the droplet itself. At low temperature the adatom diffusion length $\ell _D$ is small, compared to the average terrace width $W_T$. So, the presence of the ES barrier, which limits the adatom diffusion perpendicularly to the steps, is not affecting the adatom capture kinetics. The actual environment of the nucleation site is not playing a fundamental role at low *T*, and a unimodal behavior is predicted. As the temperature increases, the diffusion length increases until $\ell _D > W_T$. However, the presence of a sizable ES barrier limits the adatom CZ of each droplet within the terrace where the droplet is located. This introduces a broadening effect in the island size which has a marked local dependence, as it depends on the actual terrace width on which the droplet nucleated. In particular, the origin of the bimodal distribution of height is related to the observation that the droplets, nucleated at terrace edges, can collect adatoms from the two contiguous terraces. As the temperature further increases, the droplet CZ size is no more affected by the presence of finite width terraces, as the ES barrier at the step edges can be overcome by adatoms. This makes the CZ size independent from the local environment, thus restoring the unimodal size distribution of the island size.

## Conclusions

Ga droplet nucleation dynamics on singular GaAs(111)A substrates, that we analyzed through the CZD approach^28,29^, is characterized by a small critical nucleus size *i* = 1-2 atoms. This finding supports the recent critical cluster determination by Ohtake et al.^22^ based on the droplet density dependence on metal flux. It shows that lower reactivity of GaAs(111)A, respect to GaAs(001), permits the stabilization of smaller predicted magic Ga cluster, constituted by just three atoms^43^. On the contrary, larger *i* is observed on GaAs(001) substrates^43,44^. The single activation energy for the droplet density dependence on the temperature ($E_{a} = 1.13\pm 0.23\,\hbox {eV}$) is observed in the whole measured range. At low and high temperature the spatial distribution of the droplets on the surface is remarkably regular, with a HS index $I_{HS} = 0.35$, thus approaching that of the hexagonal lattice ($I_{HS} = 0.14$). The average excluded area around each island is highly symmetrical, with a nearly hexagonal shape with the vertices in $<2\,\overline{1}\,\overline{1}>$ directions. The shape of the excluded zone suggests that $<2\,\overline{1}\,\overline{1}>$ directions are preferable for Ga adatom diffusion on GaAs(111)A. This observation is in agreement with Ga-vacancy (2$\times$2) surface reconstruction of GaAs(111)A^30,31^.

At intermediate temperatures (350–$400^{\circ }\hbox {C}$) we observe the bimodal size distribution of GaAs islands, connected with a sharp increase of the disorder of the island sites, as measured by the HS index. At $T = 400^{\circ }\hbox {C}, 0.45< I_{HS} < 0.55$, with a marked dependence on the sample site. We attributed such behavior to the influence of local surface morphology which is sampled by the island CZ during its growth. Wide terraces (tens of nm wide) are observed on the singular GaAs(111)A surface and related to the presence of large and flat hillocks generated by the stacking faults^16,40,41^. The bimodal size distribution is attributed to the presence of the strong ES barrier at the terrace step edges^16^, which limits Ga adatom diffusion on the terraces, thus inducing strong differences in the CZ size for droplets are nucleated on the terraces or at the terrace step edges. The same effect is responsible of the increase of $I_{HS}$ index and the local variation.

## Data Availability

The datasets generated during and/or analysed during the current study are available from the corresponding author on reasonable request.
